# Exposure to Pesticides and Breast Cancer in an Agricultural Region in Brazil

**DOI:** 10.1021/acs.est.3c08695

**Published:** 2024-06-06

**Authors:** Carolina Panis, Luciano Zanetti Pessoa Candiotto, Shaiane Carla Gaboardi, Géssica Tuani Teixeira, Fernanda Mara Alves, Janaína Carla da Silva, Thalita Basso Scandolara, Daniel Rech, Susie Gurzenda, Jamie Ponmattam, Joyce Ohm, Marcia C. Castro, Bernardo Lemos

**Affiliations:** † Laboratory of Tumor Biology, State University of Western Paraná, UNIOESTE, Francisco Beltrão, Paraná 85605-010, Brazil; ‡ Department of Environmental Health, Harvard TH Chan School of Public Health, Boston, Massachusetts 02115, United States; § Territorial Studies Group (GETERR), State University of Western Paraná, UNIOESTE, Francisco Beltrão, Paraná 85605-010, Brazil; ∥ Catarinense Federal Institute, Campus Ibirama, Ibirama, Santa Catarina 89140-000, Brazil; ⊥ Department of Global Health and Population, Harvard TH Chan School of Public Health, Boston, Massachusetts 02115, United States; # R Ken Coit College of Pharmacy, Department of Pharmacology and Toxicology, The University of Arizona, Tucson, Arizona 85721, United States; ∇ Department of Cancer Genetics and Genomics, Roswell Park Cancer Institute, Buffalo, New York 14263, United States; ○ Department of Biochemistry and Molecular Medicine, Universite de Montreal, Montreal H3C 3J7, Canada; ◆ Instituto Nacional de Câncer, INCA, Rio de Janeiro 20231-050, Brazil

**Keywords:** pesticides, breast cancer risk, breast metastasis

## Abstract

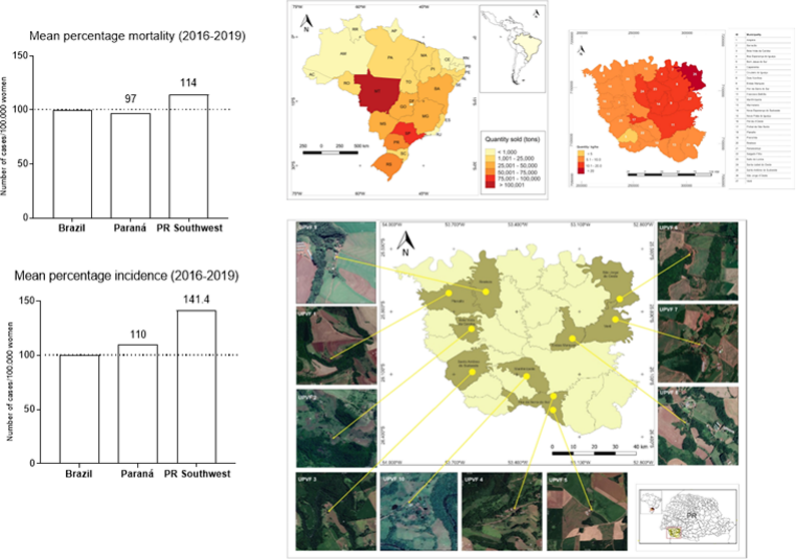

Rural workers are disproportionally exposed to pesticides and might be at an increased risk of developing chronic diseases. Here, we investigated the impact of pesticide exposure on breast cancer (BC) risk and disease profile in rural female workers. This is a case-control study that prospectively included 758 individuals. The study was conducted in the Southwest region of Paraná state in Brazil, a region characterized by family-based agriculture and intensive use of pesticides. We found that this region has a 41% higher BC diagnosis rate and 14% higher BC mortality rate than the mean rates in Brazil, as well as a pesticide trade volume about 6 times higher than the national average. We showed substantial exposure in this population and found that even women who did not work in the fields but performed equipment decontamination and clothes washing of male partners who worked in the fields had urine samples positive for glyphosate, atrazine, and/or 2,4-D. The crude association showed a significantly higher risk of BC among women exposed to pesticides (OR: 1.58, 95% CI 1.18–2.13). Adjusted analyses showed a lower and nonstatistically significant association (OR: 1.30, 95% CI 41 0.87–1.95). Stratification on disease profile showed a significantly higher risk of lymph node metastasis (adjusted OR: 2.19, 95% CI 1.31–3.72) in women exposed to pesticides. Our findings suggest that female populations exposed to pesticides are at a higher risk of developing BC with a more aggressive profile and draw attention to the need to monitor rural populations potentially exposed to pesticides in the field or at home.

## Abstract 2

In this study, we demonstrated that exposure to pesticides increases the risk for breast cancer development and promotes a more aggressive metastatic disease.

## Introduction

Human exposure to pesticides has been linked to age-associated diseases, such as cancer,^1−4^ particularly for chronically exposed rural workers.^5−8^ Cancers of the thyroid,^9^ skin,^10^ kidneys,^11^ lymph nodes,^12^ larynge,^13^ lung,^14^ colon,^15^ and prostate^16^ have been reported to have a significantly higher incidence in pesticide-spraying farmers.

Brazil is one of the top global consumers of pesticides.^20^ Particularly, the Southwest region of the state of Paraná is one of the main pesticide trading regions in Brazil. The region is characterized by widespread family farming and intensive use of agrochemicals, such as glyphosate, atrazine, and 2,4-D.^22^ In some areas with a predominance of family farming, women play a leading role in the field, while in others, they maintain domestic activities that support family members who work in the field.^17^ In the Southwest region of Paraná State and other areas in Brazil, handling pesticides in rural work is considered a predominantly male activity.^18^ Nevertheless, women are not exempt from the risk of direct exposure.^19^ This is because women could be exposed through their unprotected manipulation of pesticides (e.g., via preparation and dilution of pesticides), as well as through equipment decontamination and handling of contaminated clothing. Indeed, women’s participation in rural work through pesticide manipulation and equipment decontamination has been well documented.^21^ However, these routes of exposure that occur within the household have often been dismissed and neglected. The outcome is that limited attention has been given to the consequences of exposure of female family members not engaged in pesticide application.

Glyphosate, atrazine, and 2,4-D are reported as endocrine disruptors,^23−26^ a key mechanism linked to the development of hormone-dependent cancers, such as breast cancer (BC).^27−33^ Increased risk for BC has been suggested in wives of farmers working with a wide range of pesticides, such as fungicides,^34^ organophosphates,^35^ and organochlorines.^36^ However, in such studies, female exposure has often been dismissed. Moreover, the extent of their contamination is not typically documented, with information about the presence of pesticides in women’s blood/urine often lacking. All in all, data regarding women’s exposure to pesticides at home either via pesticide manipulation or via clothing and equipment decontaminating are scarce. Data concerning disease profiles in women chronically exposed to pesticides and the correlation between exposure and disease outcomes are also similarly limited.

Here, we report the impact of pesticide exposure on women exposed to pesticides via unprotected pesticide manipulation, handling of contaminated clothing, and equipment. We also report the potential implications for pesticide exposure to BC risk. We investigated the relationship between pesticide exposure and BC by documenting women exposure to glyphosate, atrazine, and 2,4-D. We evaluated the odds ratios associated with pesticide exposure and compared exposed and unexposed patients to address the clinicopathological profile of pesticide-exposed BC patients.

## Methods

This a case-control study that prospectively included a total of 758 individuals. This study collected individual and clinicopathological data from women at a public Oncology Hospital located at the eighth Health Regional of Paraná state (Hospital de Cancer de Francisco Beltrão, Ceonc) from January 2016 to December 2019. This region comprises an area of 7768 km^2^ and a total of 27 municipalities with approximately 330,000 inhabitants. The economy is based on small-scale farms and conventional rural activities, mainly grain crops (soy, corn, and wheat), milk cattle, and chicken livestock, in which pesticide spraying is largely done manually (with spraying machines carried in workers’ backs).

The Institutional Ethics Committee on Research of the State University of Western Paraná, CAAE 35524814.4.0000.0107, approved this research. All participants signed written informed consents and were informed about the research aims. All women screening for breast cancer were invited to join the study. Based on the analysis of the biopsies by a pathologist, women were categorized according to the presence of breast cancer or benign lesions. Among those diagnosed with BC, the study included women carrying operable tumors (TNM stage II). We included a total of 728 patients for the BC risk study and 30 individuals for the pesticide contamination study.

The study included the following information:

(i) Publicly available epidemiological data concerning breast cancer incidence and mortality in the Southwest region of Paraná during the period (2016–2019), as well as information regarding pesticide trade for each Brazilian state;
(ii) Novel pesticide contamination data of urine samples collected from 30 women who reported handling contaminated equipment or pesticides at home but who did not participate in pesticide spraying in the field. This assessment was aimed at understanding if this mode of contact was sufficient to generate contamination by glyphosate, atrazine, and 2,4-D;
(iii) A cancer risk analysis, which included 728 women diagnosed with breast cancer or not diagnosed with breast cancer, and who were exposed to pesticides or not exposed to pesticides; and
(iv) A clinicopathological characterization of women with breast cancer.

Epidemiological data about BC incidence and mortality rates were obtained from hospital records, the National Cancer Institute reports (INCA: https://www.inca.gov.br/publicacoes/livros/estimativa-2023-incidencia-de-cancer-no-brasil), and the Mortality Atlas (https://www.inca.gov.br/app/mortalidade). The mean pesticide trade for each Brazilian state, as well as for the 27 included municipalities, was obtained from the Brazilian Institute of Environment and Renewable Natural Resources (IBAMA: http://www.ibama.gov.br/agrotoxicos/relatorios-de-comercializacao-de-agrotoxicos) and the Control System for the Trade and Use of Pesticides in the State of Paraná (SIAGRO: https://www.adapar.pr.gov.br/sites/adapar/arquivos_restritos/files/documento/2022-05/dados_siagro_21_1.xls) databases.

Information concerning pesticide exposure was collected through individual interviews based on a validated questionnaire developed for this purpose.^21^ The study population consists of female rural residents. Women in this region do not typically participate in pesticide spraying. Exposure assessment occurred via a questionnaire that captured both modes of exposure—in the household and through pesticide spraying in the fields—and through assessment of urine samples. For the assessment of urine samples, we chose households where women did not participate in the pesticide spraying. This was a conservative choice because exposure in the household is expected to be lesser than exposure in the fields. It was important for us to assess exposure in the household because this mode of exposure has been neglected and has been suggested to be negligible. Our assessment dispels that notion and shows that even women who work within the household are at risk of significant exposure.

The questions yielded data about continuous contact with pesticides, lifetime working with pesticides, wearing of personal protection equipment (PPE) while spraying or washing/decontaminating clothes and PPE, and the use of protective gloves when doing these procedures. Based on such questions, women were considered as exposed to pesticides or not exposed to pesticides. The pesticide-exposed group was formed by rural women who positively answered pesticide-contact questions and lived at least 40% of their adult life working with pesticides. In contrast, the unexposed group was composed of women without a history of rural work or pesticide-related occupations in the household. To be most conservative during exposure assessment, we only evaluated women whose husbands or family members worked in the field but who did not themselves work in the fields. The most likely place of exposure for these women is in the household.

To investigate if pesticide exposure in these circumstances was enough to generate women’s contamination, urine samples were collected from 30 individuals (10 women + 10 husbands + 10 other family members) at the peak of pulverization of the most commercialized pesticides in Paraná state: glyphosate, atrazine, and 2,4-D. The presence of such residues was analyzed by commercial immunoassay-based kits (Abraxis) from ten distinct farms, randomly selected across the 27 municipalities. Urine samples (30–50 mL) were collected in sterile tubes from all family members who reported directly manipulating pesticides. Samples were collected from one woman and her relatives (applicators #1 and #2) until 6 h after glyphosate, atrazine, or 2,4-D spraying by not-mechanized methods, and the results were expressed as ppb (parts per billion). The applicators reported using intercostal pumps for spraying pesticides for about six consecutive hours. All exposed women reported having pesticide unprotected contact by diluting the concentrated package of the chemicals, filling the intercostal pump with the diluted pesticides, and washing/decontaminating clothes/PPE used for this end after spraying without wearing gloves or any other protection equipment. For BC risk calculation, data concerning pesticide exposure were collected from women with and without BC diagnosis.

Individual and tumor information was obtained from patient’s medical records to evaluate the relationship between pesticide exposure and clinicopathological features. Data from BC patients included age at diagnosis, menopausal status at diagnosis, body mass index values (BMI), BC molecular subtype, presence of lymphatic or distant metastasis, chemoresistance profile, and disease recurrence. Only the age at diagnosis, menopausal status, and body mass index (BMI, kg/m^2^) values were collected from patients without a BC diagnosis.

For data analysis, we stratified individual characteristics of participants into exposure groups and calculated the mean and standard deviation for continuous variables and counts with percentages for categorical variables. Means of continuous variables were compared using the Wilcoxon rank sum test and categorical variables were compared using Pearson’s χ^2^ test. We performed the same calculations for cancer characteristics among participants with breast cancer. We similarly analyzed participants stratified by breast cancer subtype.

An unstratified correlation analysis was conducted across all variables in the study. We also stratified participants by breast cancer diagnosis and exposure to pesticides. All correlations were calculated as Pearson’s correlation coefficients and analyzed for significance.

To assess the risk of breast cancer given pesticide exposure, we calculated the univariate odds ratio. We used the complete data set to calculate the odds ratio of developing breast cancer given exposure to pesticides compared to the risk of developing breast cancer among those not exposed to pesticides. Among women who developed breast cancer, we then calculated the odds ratio of specific molecular subtypes, histological grades, and other tumor markers associated with breast cancer given pesticide exposure. To assess molecular subtypes and histological grade—in addition to comparing each subtype to the most benign case (luminal A subtype and grade 1 tumors)—we calculated the odds ratio of various combinations of subtypes and histological grades.

For the clinical variables that had significant univariate odds ratios, we conducted logistic regressions to assess their association with pesticide exposure, adjusted for breast cancer risk factors of age, menopause status, and BMI (body mass index). Raw data for each patient are provided in Supporting Table 1.

## Results

### High Rates of BC Incidence and Mortality

**Figure 1:**
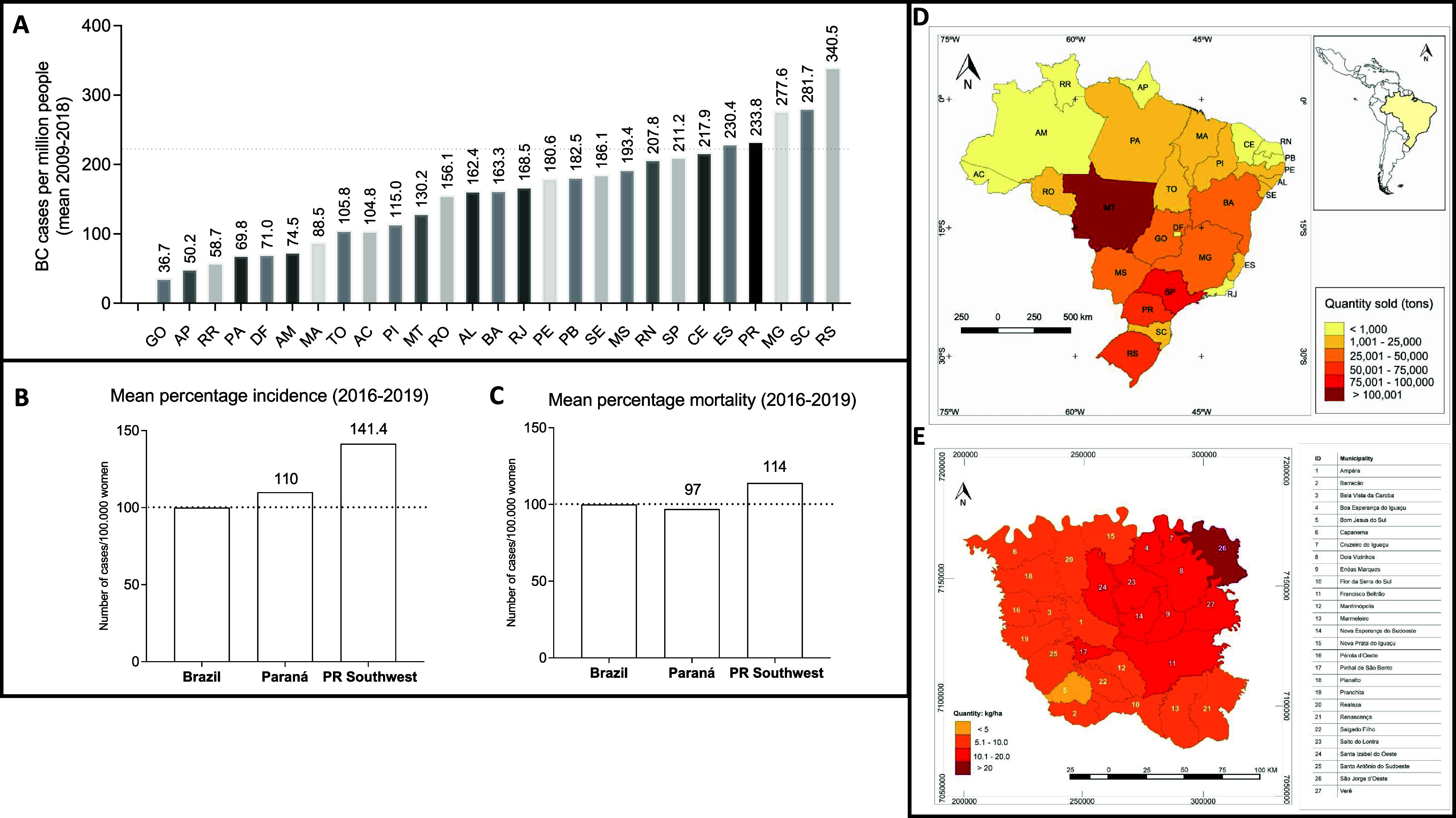
Breast cancer epidemiology and pesticide trade in Brazil and Paraná state. Mean breast cancer (BC) cases among the 27 Brazilian States from 2009 to 2018. The black bar highlights Paraná state as the fourth state with higher BC incidence. The dotted line represents the mean of BC cases in Brazil (per million people). The comparative mean percentage incidence (B) and mortality (C) are presented for Brazil, Paraná state, and the Paraná Southwest region from 2016 to 2019 (the period of data collection for this study). (D) Map of Brazil showing the mean pesticide trade for each state during the 2016–2019 interval; Paraná state is the third biggest consumer of pesticides. (E) the 27 municipalities from Paraná Southwest regions were distributed according to their pesticide trade for 2016–2019; the darker the color, the larger the amount of pesticides used by the municipality. Acre—AC; Alagoas—AL; Amapá—AP; Amazonas—AM; Bahia—BA; Ceará—CE; Distrito Federal—DF; Espírito Santo—ES; Goiás—GO; Maranhão—MA; Mato Grosso—MT; Mato Grosso do Sul—MS; Minas Gerais—MG; Pará—PA; Paraíba—PB; Paraná—PR; Pernambuco—PE; Piauí—PI; Roraima—RR; Rondônia—RO; Rio de Janeiro—RJ; Rio Grande do Norte—RN; Rio Grande do Sul—RS; Santa Catarina—SC; São Paulo—SP; Sergipe—SE; Tocantins—TO.

A comparative analysis concerning mean BC incidence and mortality (Figure 1A–C) shows that the Southwest region of Paraná presents higher rates of BC incidence and mortality relative to the state of Paraná as a whole or Brazil. Specifically, the Southwest of Paraná has a 31% higher incidence than Paraná state and 41% higher incidence than Brazil, as well as 17% higher mortality than Paraná state and 14% higher mortality than Brazil.

During the documented period (2016–2019), Paraná occupied the third position in the rank for pesticide trade in the country with 61,712 tons of pesticides traded during the period (Figure 1C). This is only behind the states of Mato Grosso and São Paulo with 106,632 tons and 82,286 tons of pesticides traded, respectively. The mean pesticide trade for Brazil was 590,670 tons in the same period. Due to its agriculture-based economy, the Southwest region of Parana has a per capita consumption of pesticides that is significantly higher than the Brazilian average of about 6.7 kg/hectare (Figure 1D). Importantly, the region is characterized by small farms, in which the extensive use of nonmechanized spraying of pesticides in family-based agriculture is predominant.

### Pesticide Contamination in the Female Study Population Occurs during Unprotected Equipment Decontamination and Clothes Washing

**Figure 2:**
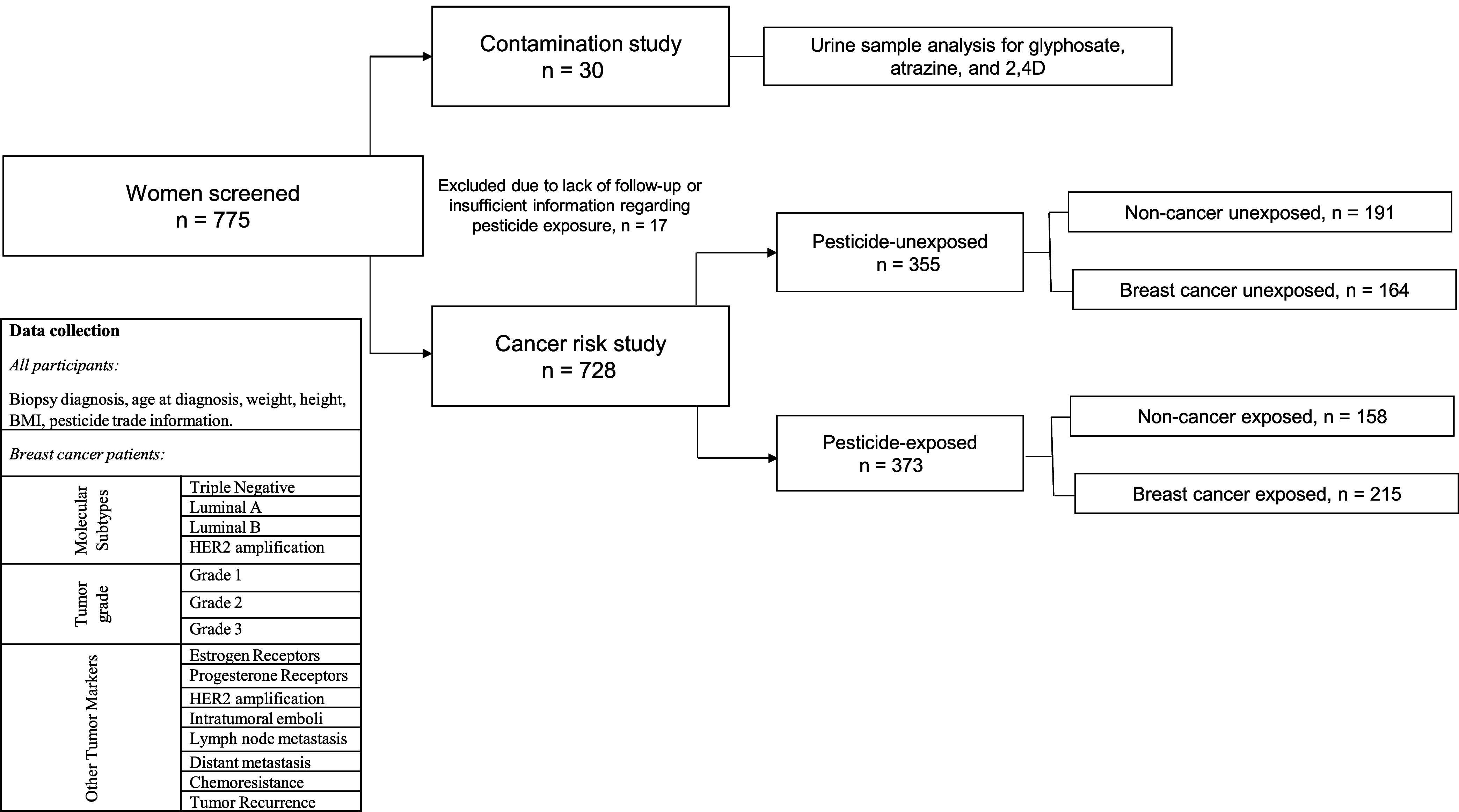
Design of the study. A total of 775 women, who attended a public hospital (Francisco Beltrão Cancer Hospital—Ceonc, Francisco Beltrão—Paraná, Brazil), were screened. After signing consent forms, patients were interviewed to obtain their pesticide exposure profiles. Seventeen women were excluded from the study due to THE lack of follow-up or insufficient information regarding pesticide exposure. Thus, 758 women were included in the study. Urine samples from 30 women were collected in the exposure assessment (contamination) study, aiming to measure glyphosate, atrazine, and 2,4-D residues. For the cancer risk study (*n* = 728), a total of 373 women were characterized as exposed and 355 as unexposed. Based on the biopsy result, patients were categorized into four groups: Women exposed to pesticides diagnosed with breast cancer (*n* = 215), women exposed to pesticides without breast cancer (*n* = 158), women not exposed to pesticides diagnosed with breast cancer (*n* = 164), and women not exposed to pesticides without breast cancer (*n* = 191). Data obtention included patient characteristics (age at diagnosis, weight, height, body mass index, menopausal status, disease onset), tumor features molecular subtypes, hormonal receptors status, human epidermal growth factor 2 receptor amplification (HER2), the presence of tumor emboli or lymph nodal metastasis, distant metastasis, chemoresistance, and tumor recurrence.

Given the region’s characteristics, where male family members are primarily engaged in pesticide application, we characterized pesticide exposure in women who are not working in the field. As shown in Figure 2, a total of 10 rural properties located in 9 municipalities from Paraná state were included. We selected farms that had a family member with BC diagnosis. BC patients from the selected properties live in households, where the male partner was primarily occupied in agriculture and had a history of pesticide use over several years. Data from the exposure characterization interview (Figure 2, Box A) showed that most patients were in continuous contact with pesticides at least once a week (57%) and spent more than 40% of their life working with pesticides (53%).

Remarkably, about 95% of exposed patients who did not work in the fields reported that they did not wear protective gloves during clothes/PPE decontamination. We therefore suspect that this might be a significant route of exposure in this population. Indeed, because of their exposure profile, we analyzed urine samples from women after unprotected pesticide manipulation and clothes/PPE decontamination. Results of urine sample analyses obtained from exposed women are shown in Figure 2, Box B.

Approximately 53% of the samples had some of the three pesticides detected (33.3% of BC patients, 43.3% for applicators #1 and #2). Glyphosate was the most often detected pesticide (at concentrations ranging from 0.25 to 2.12 ppb), followed by atrazine (1.9 to 5.9 ppb) and 2,4-D (29.3 to 80.8 ppb). The simultaneous detection of the three pesticides was found in Farms #3 and #4, where the families reported spraying pesticides for more than 6 h/daily. A urine sample from a BC patient living in farm #3 had higher levels of pesticide exposure than those found in the family applicators for all three tested pesticides. In farm #6, only the BC patient had a positive sample (0.91 ppb for glyphosate). Both women from farms #3 and #6 reported decontaminating the family’s clothes and PPE after pesticide spraying. Urine samples from BC patients living in farm#10 and #4 were the only negative for pesticides when family members were positive.

### Exposure to Pesticides and a Higher Risk of Developing BC and Disease Metastasis

Considering the co-occurrence of high BC rates and elevated pesticide use in the Southwest region of Paraná state, we investigated if there was a relationship between pesticide exposure and BC risk or disease profile. To address the issue, we examined 728 women, who went to a public hospital for breast screening between January 2016 and December 2019. Women were divided into four groups based on their exposure to pesticides as well as biopsy results: exposed with BC (*n* = 215), exposed without BC (*n* = 158), unexposed with BC (*n* = 164), and unexposed without BC (*n* = 191). Medical records were assessed to obtain data about a patient’s characteristics and tumor features.

Table 1 shows the individual characteristics of women included in the study. Women without cancer had a mean age at diagnosis of 40.8 years, and patients having BC showed a mean age at diagnosis of 55.7 years. BMI was 26.6 mg/m^2^ in no cancer group and 28.0 kg/m^2^ for BC patients. The mean volume of pesticides traded (2011–2016) was 281.6 tons in the municipalities from no cancer women and 276.9 tons in the municipalities from BC patients.

**Table 1:** Individual Characteristics of Breast Cancer Cases and Controls^a^^,^^b^

	no cancer	breast cancer
individual characteristics		
age at diagnosis (years)	40.8 (16.6)	55.7 (12.8)
weight (kg)	70.3 (14.8)	71.6 (14.1)
height (m)	1.63 (0.0656)	1.60 (0.0676)
BMI (kg/m^2^)	26.6 (5.09)	28.0 (5.49)
municipality information		
mean volume of pesticides traded between 2011 and 2016 (tons)	281.6 (113.7)	276.9 (126.2)

a Proportions are presented for categorical variables with percentages in parentheses.

b Means are presented for numerical variables with standard deviations in parentheses.

Regarding pesticide exposure (Table 2), in women without BC, the mean age of the negative diagnosis was 38.06 years for unexposed controls and 44.22 years for the exposed ones (*p* < 0.001). The mean BMI was 26.17 kg/m^2^ for the unexposed and 27.01 for the exposed ones (*p* = 0.072). For the BC groups, both unexposed and exposed women had the same mean age at diagnosis (55.67 years, *p* > 0.9) and similar BMI (28.19 and 27.85 kg/m^2^, respectively, *p* = 0.8). BC patients from both unexposed and exposed groups had similar proportions of early disease onset (41 and 38%, respectively, *p* = 0.6) and menopausal status (71 and 68%, respectively, *p* = 0.7).

**Table 2:** Individual Characteristics of Women Included in the Study and Municipatlity Information Concerning Pesticide Trade^a^

	no cancer			breast cancer		
	unexposed *n* = 191	exposed *n* = 158	*p*-value^c^^,^^d^	unexposed *n* = 164	exposed *n* = 215	*p*-value^c^^,^^d^
individual characteristics						
age at diagnosis	38.06 (17.04)	44.22 (15.55)	<0.001^e^	55.67 (12.91)	55.67 (12.83)	>0.9
early onset (<50 years)				64 (41%)	81 (38%)	0.6
menopause at diagnosis				108 (71%)	141 (68%)	0.7
weight (kg)	69.81 (15.85)	70.85 (13.69)	0.3	71.84 (14.27)	71.35 (13.95)	0.7
height (m)	1.63 (0.07)	1.62 (0.06)	0.8	1.60 (0.07)	1.6 (0.06)	>0.9
BMI (kg/m^2^)	26.17 (5.24)	27.01 (4.91)	0.072	28.19 (5.92)	27.85 (5.15)	0.8
municipality information^b^						
mean volume of pesticides traded (tons)	267.74 (114.67)	287.47 (112.57)	0.12	284.17 (124.43)	271.64 (127.56)	0.12

a Proportions are presented for categorical variables with percentages in parentheses. Means are presented for numerical variables with standard deviations in parentheses.

b Municipality information is averaged and weighted by the number of patients from each municipality.

c Wilcoxon rank sum test; Pearson’s χ^2^ test.

d We use the Yates continuity correction when estimating *p*-values comparing the proportion of exposed vs unexposed to avoid overestimating the significance of these small proportions. (1).

e *p* < 0.05.

Women exposed to pesticides had a higher risk of developing BC than unexposed controls (OR crude 1.58, CI: 1.18–2.13 for all cases, 1.51, CI: 1.05–2.17 for complete cases, and OR adjusted 1.30, CI 0.87–1.95, Table 3). The mean volume of pesticide traded in the study period was similar across all groups and ranged from 267.74 tons in the localities of unexposed women without BC to 287.47 tons in the localities of exposed women without breast cancer (*p* = 0.12). Similar profiles were observed for the tumor molecular subtype in unexposed and exposed BC patients (Table 4).

**Table 3:** Counts and Odds Ratios of Breast Cancer by Exposure

			odds ratio (95% CI)	
	exposed	unexposed	crude	adjusted^b^
all cases				1.30 (0.87–1.95)
breast cancer	215	164	1.58 (1.18–2.13)	
no breast cancer	158	191		
complete cases^a^				
breast cancer	182	139	1.51 (1.05–2.17)	
no breast cancer	86	99		

a Cases with no missing responses for age, BMI, and menopause at diagnosis.

b Adjusted for age, BMI, and menopause at diagnosis.

**Table 4:** Counts and Odds Ratios of Breast Cancer Molecular Subtypes by Exposure

	triple negative	luminal A	luminal B	HER2-amplified
exposed	48	61	69	26
unexposed	30	52	48	19
odds ratio (95% CI)^a^				
crude	1.22 (0.73–2.06)	0.796 (0.51–1.25)	1.08 (0.69–1.69)	0.999 (0.53–1.90)
adjusted^b^	1.35 (0.76–2.43)	0.683 (0.41–1.13)	1.07 (0.66–1.74)	1.16 (0.59–2.34)

a ORs are calculated as given molecular subtypes vs all other subtypes.

b Adjusted for age, BMI, and menopause at diagnosis.

Exposed BC patients presented more lymphatic metastases than the unexposed group (*p* = 0.015). Similar profiles were observed when comparing chemoresistance (*p* = 0.5) and disease recurrence (*p* = 1). A multivariate analysis of the association between lymph node metastasis with pesticide exposure, adjusting for breast cancer risk factors of age, menopause status, and body mass index (BMI), showed that the risk for being exposed and having lymph nodal metastasis is 2.19 (CI: 1.31–3.72, Table 5).

**Table 5:** Clinicopathological Characterization of Patients with Breast Cancer Occupationally Exposed or Not to Pesticides^a^

		unexposed *n* = 164	exposed *n* = 215	*p*-value^b^^,^^c^
cancer Information^b^				
lymphatic metastasis				0.015^f^
	no	96 (74%)	109 (61%)	
	yes	33 (26%)	71 (39%)	
	na	35	35	
chemoresistance				0.5
	no	138 (85%)	171 (81%)	
	yes	25 (15%)	39 (19%)	
	na	1	5	
recurrence				1
	no	130 (89%)	170 (89%)	
	yes	16 (11%)	22 (11%)	
	na	18	23	
multivariate analysis for lymphatic metastasis^d^			odds ratio (95% CI)	
crude			1.86 (1.14–3.07)	
adjusted^e^			2.19 (1.31–3.72)^f^	

a NA = data not available.

b We use the Yates continuity correction when estimating p-values comparing the proportion of exposed vs unexposed to avoid overestimating the significance of these small proportions (1).

c Pearson’s Chi-squared test. 3. Multivariate analysis of the association between breast cancer and lymph node metastasis with pesticide exposure, adjusting for breast cancer risk factors of age, menopause status, and body mass index (BMI).

d Adjusting for breast cancer risk factors of age, menopause status, and body mass index (BMI).

e Adjusted for age, BMI, and menopause at diagnosis.

f *p* < 0.05.

**Figure 3:**
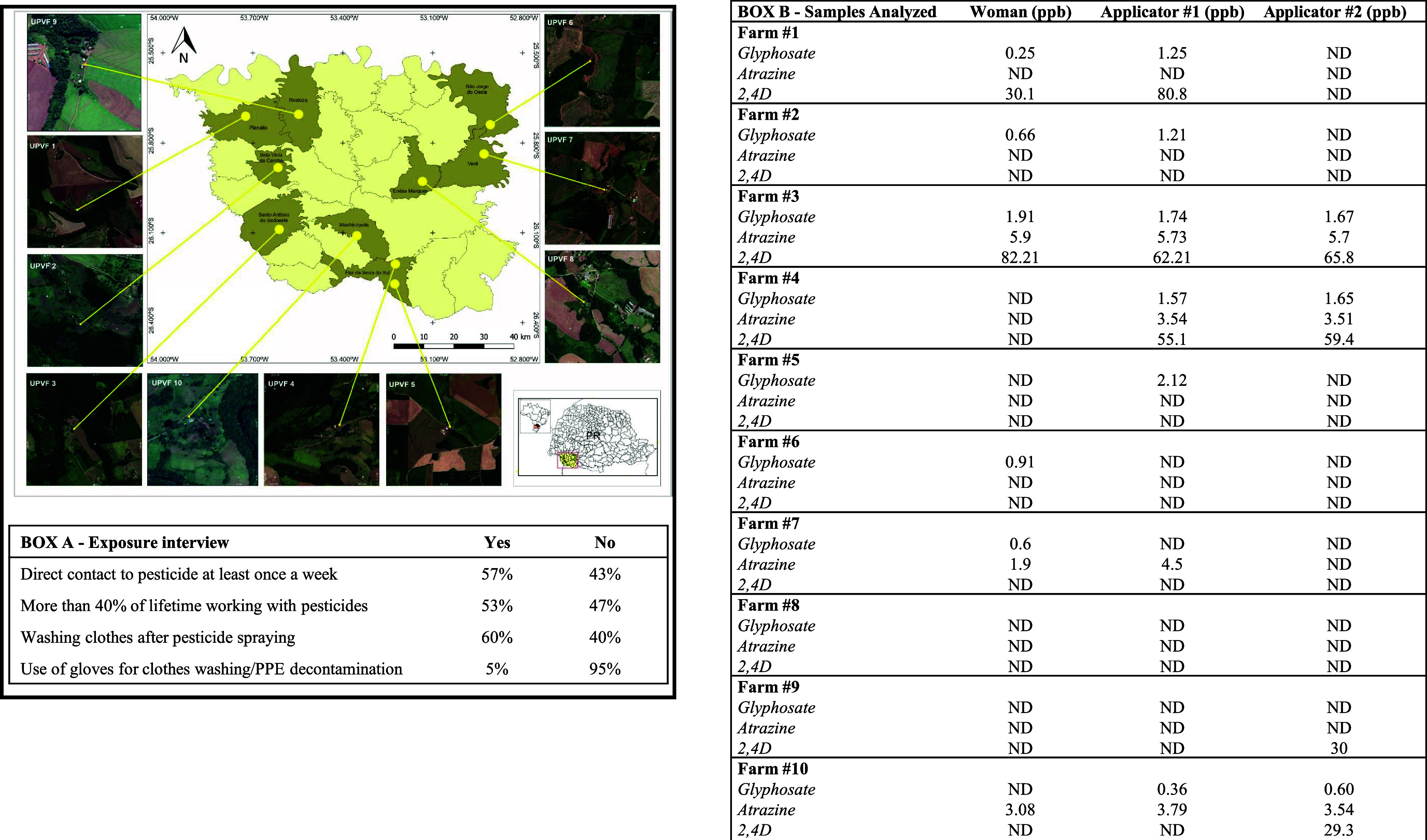
Pesticide contamination study. A total of 10 farms were chosen to conduct the exposure assessment (contamination) study, as shown on the map. This step was carried out during the peak of pulverization of the three most traded pesticides in the region (glyphosate, atrazine, and 2,4-D). An interview was conducted to obtain their occupational exposure profile to pesticides, and the results are shown in Box A. Urine samples were collected to evaluate pesticide concentration after its pulverization, and the results are shown in Box B. ND = not detected, ppb = parts per billion.

**Figure 4:**
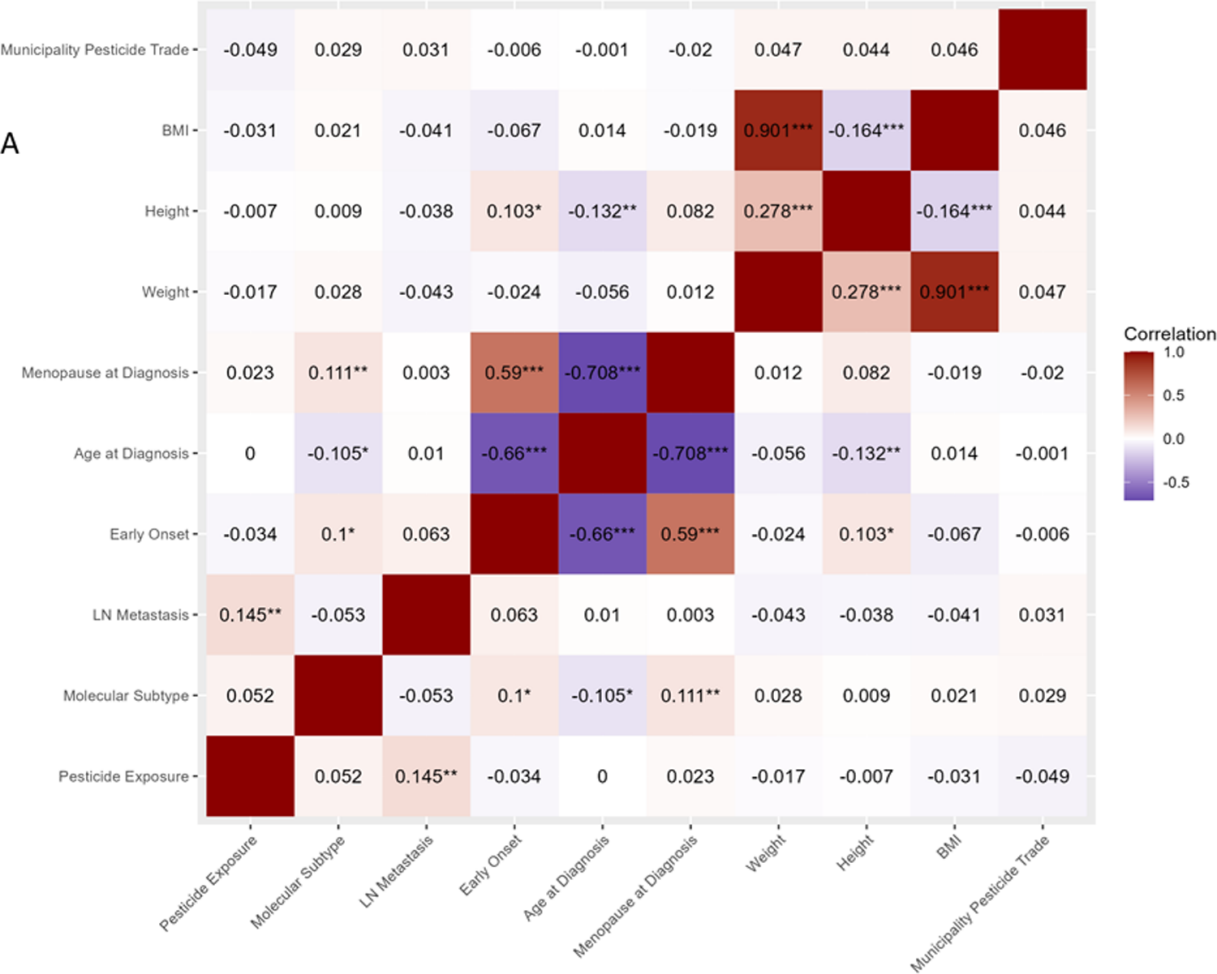
Correlation matrix of select variables for all breast cancer patients. BMI = body mass index, LN = lymph nodal. * *p*-value < 0.1, **p-value < 0.05, and ***p-value < 0.01.

Spearman correlation analysis among clinicopathological parameters from all BC patients showed a significant positive correlation between pesticide exposure and the presence of lymph nodal metastasis (Figure 4, *R* = 0.145, *p* < 0.05) (Figure 3). Other statistically significant correlations were clinically expected (Figure 4).

## Discussion

The role of women in family-based agriculture is particularly underappreciated in regions where fieldwork is typically conducted by male family members.^37^ Because of this, studies focusing on women’s health issues due to pesticide exposure are rare in these settings. In the present study, we detailed how pesticide exposure occurred in women not engaged in pesticide application and investigated their increased risk for BC development and metastasis. We reported that women exposed to pesticides have higher breast cancer risk than the unexposed ones. Also, urine analysis for glyphosate, atrazine, and 2,4-D residues showed that the unprotected manipulation of pesticide-containing items results in women’s contamination. Furthermore, pesticide exposure significantly correlated to disease metastasis.

The main route of pesticide exposure was unprotected PPE decontamination and washing of contaminated clothes after pulverization by family members. Exposure to pesticides occurs mainly due to their misperceptions about the hazardous risk of such chemicals and the wrong notion that PPE decontamination and handling of clothing is not a significant source of exposure. Further, the lack of adequate spaces to manage contaminated equipment and low adherence to good pesticide management practices are also reported as primary sources of widespread female contamination in these small-scale family owned properties.^19^

Studies of female exposure to pesticides have mainly focused on reproductive and hormonal health and include outcomes, such as changes in placenta,^38^ female infertility,^39^ and spontaneous abortion occurrence.^40^ Previous research in Brazil has considered that women were minimally exposed, by virtue of their living on farms or having limited contact with pesticides through their husbands who engaged in the spraying of pesticides; however, no research to date has evaluated women pesticide exposure in the household and the implications for disease risk profile. Our analysis indicates that women exposure to pesticides is substantial even when they are not directly engaged in pesticide spraying and that the implications of exposure to cancer risk are not negligible. This is because exposure levels through contact with contaminated equipment, clothes, and pesticide dilution at home can be substantial. Further, little attention has been given to other chronic, life-threatening pathologies such as BC. The gap is particularly relevant because BC is the leading cause of women’s death worldwide, a cancer for which most cases result from environmental factors and life habits, with a minority of cases attributable to heritable high-risk genes.^41^ Evidence addressing BC risk and pesticide exposure for potentially exposed women is not well documented. Indeed, data concerning specific pesticide exposures or pesticide measurement in biological samples from female rural workers are typically unavailable. We show that BC risk for exposed women is 32% higher compared to unexposed ones in the crude analysis. This risk was lower and not significant when adjusting for age, BMI, and menopause at diagnosis (adjusted OR: 1.30, IC_95_: 0.87–1.95).

Pesticides may pose a substantial risk for BC development. Current evidence is based, for instance, on case-control studies characterizing female environmental contact with organochlorines.^42−46^ Risk assessment studies about occasional pesticide exposure and BC development have also shown a positive association in multiple populations, with hazard risks reaching more than 4 times in some cases but not others.^7,25,34,43,45,47,48^ This variability may result from various factors, such as epidemiological studies that analyze the overall population with individuals who are occasionally/accidentally exposed to pesticides versus target populations with high exposure, such as rural workers. The pesticide residues found in the present study were very similar to others identified in surveys of urine samples in populations worldwide. Prior analyses include surveys in countries with rigorous pesticide use restrictions, such as those from the European Union,^48−51^ and very permissive nations, such as Brazil^52^ and the US.^53−55^ Detected levels are reported in the magnitude of ppb, though they could be conservatively viewed as underestimates of exposure levels due to the short half-life of such substances in the body. These findings reinforce that all sources (food, drinking water, occupational) can lead to pesticide contamination, even though it needs to be clarified how to define the impact of such concentrations on people’s health.

Glyphosate is the most used herbicide and is classified by International Agency for Research on Cancer (IARC) as a probable human carcinogen. The presence of the glyphosate metabolite aminomethylphosphonic acid (AMPA) in women’s urine samples has been associated with BC development.^56^ Plausible carcinogenic mechanisms for this substance include changes in DNA methylation^57^ and alterations in the estrogen pathway^58^ that can occur under environmentally relevant concentrations.^59^ 2,4-D is classified by IARC as a possible human carcinogen. Its carcinogenic-linked mechanisms include the generation of oxidative stress^61^ and endocrine disruption.^62^ One study in California showed an increased risk for BC development in 2,4-D-exposed female workers.^60^ Furthermore, it has been suggested that 2,4-D has potentially genotoxic effects when coexposed with glyphosate.^63^ Finally, atrazine is classified by IARC as lacking a carcinogenic risk designation for humans. However, evidence suggests that atrazine has an endocrine-disrupting mechanism in both normal^64^ and cancerous breast cells,^65,66^ though it showed no association with BC risk in human environmental exposure studies.^67−69^ No data regarding female exposure to atrazine was found in the literature.

Investigating environmental factors that enhance metastasis occurrence is of great value to preventive medicine and public health. A major finding of our study is that pesticide-exposed women diagnosed with BC have a 54% higher risk of metastasis than unexposed women with BC. It is relevant because metastases are responsible for most cancer-related deaths.^70^ The metastatic process is characterized by a series of events triggered by the detachment of cancer cells from the primary tumor, which join the lymphatic and circulatory systems to invade distant organs.^71^ Interestingly, we also observed that exposed BC patients exhibit more lymph nodal metastasis than unexposed BC patients, and pesticide exposure correlates positively to lymph nodal invasion. These findings reinforce the plausible role of chronic pesticide exposure on disease aggravation. It is essential to highlight that lymph nodal-positive women are submitted to cytotoxic chemotherapy protocols that, despite mitigating BC cells, have deleterious effects on noncancerous cells. It means that BC women undergoing chemotherapy are at risk for developing drug-related toxicities that can have poor outcomes and even death.

All in all, we suggest that BC metastases may result from a series of events triggered by chronic pesticide exposure. While the direct effect of glyphosate or 2,4-D on metastasis is not reported, it has been suggested that atrazine has the potential to promote metastasis in ovarian and liver cells.^72,73^ However, no data is available for BC. Nevertheless, chronic exposure to pesticide mixtures may collectively affect BC behavior. For example, hormones of the circadian cycle are suggested as keys to promoting the dissemination of circulating tumor cells in BC^74^ and are also reported to be dysregulated during pesticide exposure.^75^ Furthermore, the relationship between pesticide exposure and metastasis might affect specific groups of BC patients and influence their risk for disease recurrence and death. Additional mechanisms linking pesticide exposure to tumor metastasis include depletion of antitumor proteins, such as interleukin 12, and augmented expression of protumoral molecules, such as CLTA-4 and TGF-β1.^76^ These mechanisms are not reported in unexposed BC women and are suggested as a worse prognosis signature linked to pesticide exposure.

This study has some limitations. A potential selection bias for the study is that the control group in the risk analysis came from the hospital-attended population and not from the general population. Also, the age at diagnosis and the menopausal status could be considered as risk factors for breast cancer. Because of this, they were considered as confounding factors in the multivariate analysis.

For instance, we have not measured all potential pesticides used in the region across all women included in the study. Other pesticides reported as frequent contaminants in Paraná state include mancozeb, diuron, and legacy pollutants, such as DDT and lindane.^33^ In addition, we cannot access their correlation to clinicopathological features. Furthermore, a longer-term follow-up and monitoring would be relevant to re-evaluate the positive and negative observations. Eventually, we envision performing a 10-year follow-up in our study population to evaluate the impact of exposure on long-term disease survival and disease risk. Accordingly, no significant differences were observed regarding disease survival and chemoresistance in exposed and unexposed BC women, possibly due to the short-term follow-up of patients (5 years on average). Further, it is difficult to point out which pesticide has a major contribution to BC development and aggressiveness since patients are exposed to mixtures of such substances.

Despite those limitations, our findings caution against dismissing the pesticides studied here as plausible sources of increased BC cancer in women regularly exposed to them. The data suggest that pesticide exposure increases the risk for BC diagnosis and metastasis development in women continuously exposed. The study also highlights the educational challenge of promoting best practices for pesticide handling and equipment/clothing decontamination in rural populations with family-based agriculture.

In conclusion, this study points to glyphosate, atrazine, and 2,4-D contamination in women exposed to pesticides at home. The relationship between increased BC and metastases risk in the exposed population needs attention and would benefit from long-term monitoring of potentially exposed women populations. Our findings reinforce the concerns regarding the increase in pesticide use over the past few years and the potential risks they pose for human health, especially when considering that our population study is exposed to a cocktail of pesticides that mainly include glyphosate, atrazine, and 2,4-D. The safe use of some pesticides is a matter of concern, including the widespread contamination of family members who are not working in the field. Long-term monitoring of these populations is urgently needed. Safety assessments for substances such as glyphosate and other widely used pesticides should be ongoing to mitigate reliance on safety profiles, and practices established decades ago that are yet to be revised.

## Data Availability

Raw data are presented as a Supporting File.

## Supporting Information Available

Raw data (XLSX)

## Notes

**Ethics Approval and Consent to Participate** All ethical issues were considered in the study and are reported accordingly in the Methods Section.

## References

[ref1] International Agency for Research on Cancer IARC Working Group on the Evaluation of Carcinogenic Risks to Humans. Occupational Exposures in Insecticide Application, and Some Pesticides, 1991. https://www.ncbi.nlm.nih.gov/books/NBK499663/. PMC7682260 1688189

[ref2] Hoang T. T.; Qi C.; Paul K. C.; Lee M.; White J. D.; Richards M.; Auerbach S. S.; Long S.; Shrestha S.; Wang T.; Freeman L. E. B.; Hofmann J. N.; Parks C.; Xu C. J.; Ritz B.; Koppelman G. H.; London S. J.; Epigenome-Wide DNA Methylation and Pesticide Use in the Agricultural Lung Health Study. Environ. Health Perspect. 2021, 129 (9), 097008 10.1289/EHP8928. 34516295 PMC8437246

[ref3] Lerro C. C.; Freeman L. E. B.; Portengen L.; Kang D.; Lee K.; Blair A.; Lynch C. F.; Bakke B.; De Roos A. J.; Vermeulen R. C. A longitudinal study of atrazine and 2,4-D exposure and oxidative stress markers among iowa corn farmers. Environ. Mol. Mutagen. 2017, 58 (1), 30–38. 10.1002/em.22069. 28116766 PMC5763550

[ref4] Jacobsen-Pereira C. H.; Cardoso C. C.; Gehlen T. C.; Dos Santos C. R.; Santos-Silva M. C. Immune response of Brazilian farmers exposed to multiple pesticides. Ecotoxicol. Environ. Saf. 2020, 202, 110912 10.1016/j.ecoenv.2020.110912. 32800247

[ref5] da Silva A. M. C.; Soares M. R.; Silva N. A.; Correa M. L. M.; Machado J. M. H.; Pignati W. A.; de Souza Andrade A. C.; Galvão N. D. Environmental and occupational exposure among cancer patients in Mato Grosso, Brazil. Rev. Bras. Epidemiol. 2022, 25, e220018 10.1590/1980-549720220018.supl.1. 35766775

[ref6] Rossides M.; Kampitsi C. E.; Talbäck M.; Mogensen H.; Wiebert P.; Tettamanti G.; Feychting M. Occupational exposure to pesticides in mothers and fathers and risk of cancer in the offspring: A register-based case-control study from Sweden (1960–2015). Environ. Res. 2022, 214, 113820 10.1016/j.envres.2022.113820. 35809638

[ref7] de Graaf L.; Talibov M.; Boulanger M.; Bureau M.; Robelot E.; Lebailly P.; Baldi I.; Health of greenspace workers: Morbidity and mortality data from the AGRICAN cohort. Environ. Res. 2022, 212, 113375 10.1016/j.envres.2022.113375. 35533714

[ref8] De Roos A. J.; Schinasi L. H.; Miligi L.; Cerhan J. R.; Bhatti P.; ’t Mannetje A.; Baris D.; Benavente Y.; Benke G.; Clavel J.; Casabonne D.; Fritschi L.; Hofmann J. N.; Huynh T.; Monnereau A.; Piro S.; Slager S. L.; Vajdic C. M.; Wang S. S.; Zhang Y.; Bernstein L.; Cocco P. Occupational insecticide exposure and risk of non-Hodgkin lymphoma: A pooled case-control study from the InterLymph Consortium. Int. J. Cancer. 2021, 149 (10), 1768–1786. 10.1002/ijc.33740. 34270795 PMC10560384

[ref9] Lerro C. C.; Freeman L. E. B.; DellaValle C. T.; Andreotti G.; Hofmann J. N.; Koutros S.; Parks C. G.; Shrestha S.; Alavanja M. C. R.; Blair A.; Lubin J. H.; Sandler D. P.; Ward M. H. Pesticide exposure and incident thyroid cancer among male pesticide applicators in agricultural health study. Environ. Int. 2021, 146, 106187 10.1016/j.envint.2020.106187. 33126065 PMC10127519

[ref10] Togawa K.; Leon M. E.; Lebailly P.; Freeman L. E. B.; Nordby K. C.; Baldi I.; MacFarlane E.; Shin A.; Park S.; Greenlee R. T.; Sigsgaard T.; Basinas I.; Hofmann J. N.; Kjaerheim K.; Douwes J.; Denholm R.; Ferro G.; Sim M. R.; Kromhout H.; Schüz J. Cancer incidence in agricultural workers: Findings from an international consortium of agricultural cohort studies (AGRICOH). Environ. Int. 2021, 157, 106825 10.1016/j.envint.2021.106825. 34461377 PMC8484858

[ref11] Andreotti G.; Freeman L. E. B.; Shearer J. J.; Lerro C. C.; Koutros S.; Parks C. G.; Blair A.; Lynch C. F.; Lubin J. H.; Sandler D. P.; Hofmann J. N. Occupational Pesticide Use and Risk of Renal Cell Carcinoma in the Agricultural Health Study. Environ. Health Perspect. 2020, 128 (6), 067011 10.1289/EHP6334. 32692250 PMC7292387

[ref12] Leon M. E.; Schinasi L. H.; Lebailly P.; Freeman L. E. B.; Nordby K. C.; Ferro G.; Monnereau A.; Brouwer M.; Tual S.; Baldi I.; Kjaerheim K.; Hofmann J. N.; Kristensen P.; Koutros S.; Straif K.; Kromhout H.; Schüz J. Pesticide use and risk of non-Hodgkin lymphoid malignancies in agricultural cohorts from France, Norway and the USA: a pooled analysis from the AGRICOH consortium. Int. J. Epidemiol. 2019, 48 (5), 1519–1535. 10.1093/ije/dyz017. 30880337 PMC6857760

[ref13] Lerro C. C.; Andreotti G.; Koutros S.; Lee W. J.; Hofmann J. N.; Sandler D. P.; Parks C. G.; Blair A.; Lubin J. H.; Freeman L. E. B. Alachlor Use and Cancer Incidence in the Agricultural Health Study: An Updated Analysis. J. Natl. Cancer Inst. 2018, 110 (9), 950–958. 10.1093/jnci/djy005. 29471327 PMC6136926

[ref14] Bonner M. R.; Freeman L. E.; Hoppin J. A.; Koutros S.; Sandler D. P.; Lynch C. F.; Hines C. J.; Thomas K.; Blair A.; Alavanja M. C. Occupational Exposure to Pesticides and the Incidence of Lung Cancer in the Agricultural Health Study. Environ. Health Perspect. 2017, 125 (4), 544–551. 10.1289/EHP456. 27384818 PMC5381995

[ref15] Talibov M.; Tual S.; Morlais F.; Meryet-Figuière M.; Boulanger M.; Bouvier V.; Perrier S.; Clin B.; Baldi I.; Lebailly P.; et al. Colorectal cancer among farmers in the AGRICAN cohort study. Cancer Epidemiol. 2022, 78, 102125 10.1016/j.canep.2022.102125. 35303617

[ref16] Pardo L. A.; Freeman L. E. B.; Lerro C. C.; Andreotti G.; Hofmann J. N.; Parks C. G.; Sandler D. P.; Lubin J. H.; Blair A.; Koutros S. Pesticide exposure and risk of aggressive prostate cancer among private pesticide applicators. Environ. Health 2020, 19 (1), 30 10.1186/s12940-020-00583-0. 32138787 PMC7059337

[ref17] Bezerra G. J.; Schlinwein M. M. Family Farming as income generation and local development: an analysis in Dourados, MS, Brazil. Interações (Campo Grande) 2017, 18 (1), 3–15. 10.20435/1984-042X-2016-v.18-n.1(01).

[ref18] Dahiri B.; Martín-Reina J.; Carbonero-Aguilar P.; Aguilera-Velázquez J. R.; Bautista J.; Moreno I. Impact of Pesticide Exposure among Rural and Urban Female Population. An Overview. Int. J. Environ. Res. Public Health 2021, 18 (18), 9907 10.3390/ijerph18189907. 34574830 PMC8471259

[ref19] Mrema E. J.; Ngowi A. V.; Kishinhi S. S.; Mamuya S. H. Pesticide Exposure and Health Problems Among Female Horticulture Workers in Tanzania. Environ. Health Insights 2017, 11, 1178630217715237 10.1177/1178630217715237. 28690397 PMC5484550

[ref20] FAO The Food and Agriculture Organization of the United Nations. The Role of Women in Agriculture, 2011. file:///C:/Users/miste/Downloads/a-am307e%20(1).pdf.

[ref21] Panis C.; Gaboardi S. C.; Kawassaki A. C. B.; Dias E. C. M.; Teixeira G. T.; da Silva D. R. P.; Candiotto L. Z. P. Characterization of occupational exposure to pesticides and its impact on the health of rural women. Rev. Ciênc. Farm. Básica Apl. - RCFBA 2022, 43, e748 10.4322/2179-443X.0748.

[ref22] Gaboardi S. C.; Candiotto L. Z. P.; Ramos L. M. Perfil do Uso de Agrotóxicos no Sudoeste do Paraná. Rev. Nera 2011–2016, 22 (46), 13–40.

[ref23] Nicolella H. D.; de Assis S. Epigenetic Inheritance: Intergenerational Effects of Pesticides and Other Endocrine Disruptors on Cancer Development. Int. J. Mol. Sci. 2022, 23 (9), 4671 10.3390/ijms23094671. 35563062 PMC9102839

[ref24] Maddalon A.; Galbiati V.; Colosio C.; Mandić-Rajčević S.; Corsini E. Glyphosate-based herbicides: Evidence of immune-endocrine alteration. Toxicology 2021, 459, 152851 10.1016/j.tox.2021.152851. 34246717

[ref25] Sun H.; Shao W.; Liu H.; Jiang Z. Exposure to 2,4-dichlorophenoxyacetic acid induced PPARβ-dependent disruption of glucose metabolism in HepG2 cells. Environ. Sci. Pollut. Res. 2018, 25 (17), 17050–17057. 10.1007/s11356-018-1921-6. 29633193

[ref26] Kucka M.; Pogrmic-Majkic K.; Fa S.; Stojilkovic S. S.; Kovacevic R. Atrazine acts as an endocrine disrupter by inhibiting cAMP-specific phosphodiesterase-4. Toxicol. Appl. Pharmacol. 2012, 265 (1), 19–26. 10.1016/j.taap.2012.09.019. 23022511 PMC4181665

[ref27] Coppola L.; Tait S.; Fabbrizi E.; Perugini M.; La Rocca C. Comparison of the Toxicological Effects of Pesticides in Non-Tumorigenic MCF-12A and Tumorigenic MCF-7 Human Breast Cells. Int. J. Environ. Res. Public Health 2022, 19 (8), 4453 10.3390/ijerph19084453. 35457321 PMC9030493

[ref28] Wan M. L. Y.; Co V. A.; El-Nezami H. Endocrine disrupting chemicals and breast cancer: a systematic review of epidemiological studies. Crit. Rev. Food Sci. Nutr. 2022, 62, 6549–6576. 10.1080/10408398.2021.1903382. 33819127

[ref29] Ventura C.; Nieto M. R.; Bourguignon N.; Lux-Lantos V.; Rodriguez H.; Cao G.; Randi A.; Cocca C.; Núñez M. Pesticide chlorpyrifos acts as an endocrine disruptor in adult rats causing changes in mammary gland and hormonal balance. J. Steroid Biochem. Mol. Biol. 2016, 156, 1–9. 10.1016/j.jsbmb.2015.10.010. 26518068

[ref30] IARC International Agency for Research on Cancer. IARC Monographs on the Identification of Carcinogenic Hazards to Humans, 2022. https://monographs.iarc.who.int/agents-classified-by-the-iarc/.

[ref31] Pizzatti L.; Kawassaki A. C. B.; Fadel B.; Nogueira F. C. S.; Evaristo J. A. M.; Woldmar N.; Teixeira G. T.; Da Silva J. C.; Scandolara T. B.; Rech D.; Candiotto L. P. Z.; Silveira G. F.; Pavanelli W. R.; Panis C. Toxicoproteomics Disclose Pesticides as Downregulators of TNF-α, IL-1β and Estrogen Receptor Pathways in Breast Cancer Women Chronically Exposed. Front. Oncol. 2020, 10, 1698 10.3389/fonc.2020.01698. 32984049 PMC7483484

[ref32] Scandolara T. B.; Valle S. F.; Teixeira C. E.; Scherer N. M.; Armas E. M.; Furtado C.; Boroni M.; Jaques H. S.; Alves F. M.; Rech D.; Panis C.; Bonvicino C. R. Somatic DNA Damage Response and Homologous Repair Gene Alterations and Its Association With Tumor Variant Burden in Breast Cancer Patients With Occupational Exposure to Pesticides. Front. Oncol. 2022, 8 (12), 904813 10.3389/fonc.2022.904813. PMC9305859 35875117

[ref33] Panis C.; Candiotto L. Z. P.; Gaboardi S. C.; Gurzenda S.; Cruz J.; Castro M.; Lemos B. Widespread pesticide contamination of drinking water and impact on cancer risk in Brazil. Environ. Int. 2022, 165, 107321 10.1016/j.envint.2022.107321. 35691095

[ref34] Werder E. J.; Engel L. S.; Satagopan J.; Blair A.; Koutros S.; Lerro C. C.; Alavanja M. C.; Sandler D. P.; Freeman L. E. B. Herbicide, fumigant, and fungicide use and breast cancer risk among farmers’ wives. Environ. Epidemiol. 2020, 4 (3), e097 10.1097/EE9.0000000000000097. 32613154 PMC7289136

[ref35] Engel L. S.; Werder E.; Satagopan J.; Blair A.; Hoppin J. A.; Koutros S.; Lerro C. C.; Sandler D. P.; Alavanja M. C.; Freeman L. E. B. Insecticide Use and Breast Cancer Risk among Farmers’ Wives in the Agricultural Health Study. Environ. Health Perspect. 2017, 125 (9), 097002 10.1289/EHP1295. 28934092 PMC5915194

[ref36] Louis L. M.; Lerro C. C.; Friesen M. C.; Andreotti G.; Koutros S.; Sandler D. P.; Blair A.; Robson M. G.; Freeman L. E. B. A prospective study of cancer risk among Agricultural Health Study farm spouses associated with personal use of organochlorine insecticides. Environ. Health 2017, 16 (1), 95 10.1186/s12940-017-0298-1. 28874165 PMC5585902

[ref37] FAO The Food and Agriculture Organization of the United Nations. The Role of Women in Agriculture, 2011. https://www.fao.org/3/am307e/am307e00.pdf.

[ref38] Kumar S. N.; Vaibhav K.; Bastia B.; Singh V.; Ahluwalia M.; Agrawal U.; Borgohain D.; Raisuddin S.; Jain A. K. Occupational exposure to pesticides in female tea garden workers and adverse birth outcomes. J. Biochem. Mol. Toxicol. 2021, 35 (3), e22677 10.1002/jbt.22677. 33350548

[ref39] Hanke W.; Jurewicz J. The risk of adverse reproductive and developmental disorders due to occupational pesticide exposure: an overview of current epidemiological evidence. Int. J. Occup. Med. Environ. Health 2004, 17 (2), 223–243. 15387079

[ref40] Vazirinejad R.; jamalizadeh A.; Tajik S.; Shamsizadeh A.; et al. Occupational Exposure to pesticides and spontaneous abortion among female pistachio farmers: a case-control study. J. Occup. Health Epidemiol. 2012, 1 (2), 67–74. 10.18869/acadpub.johe.1.2.67.

[ref41] de Moura J. B.; Ghedin C. C.; Takakura É. T.; Scandolara T. B.; Rech D.; Panis C. Hereditary Breast and Ovarian Cancer Screening Syndrome Profile in Women Diagnosed with Breast Cancer from Paraná State Southwest. Rev. Bras. Ginecol. Obstet. 2021, 43 (8), 616–621. 10.1055/s-0041-1733998. 34547796 PMC10183857

[ref42] Cohn B. A.; La Merrill M.; Krigbaum N. Y.; Yeh G.; Park J. S.; Zimmermann L.; Cirillo P. M. DDT Exposure in Utero and Breast Cancer. J. Clin. Endocrinol. Metab. 2015, 100 (8), 2865–2872. 10.1210/jc.2015-1841. 26079774 PMC4524999

[ref43] Miao Y.; Rong M.; Li M.; He H.; Zhang L.; Zhang S.; Liu C.; Zhu Y.; Deng Y. L.; Chen P. P.; Zeng J. Y.; Zhong R.; Mei S. R.; Miao X. P.; Zeng Q. Serum concentrations of organochlorine pesticides, biomarkers of oxidative stress, and risk of breast cancer. Environ. Pollut. 2021, 286, 117386 10.1016/j.envpol.2021.117386. 34051689

[ref44] Wolff M. S.; Toniolo P. G.; Lee E. W.; Rivera M.; Dubin N. Blood levels of organochlorine residues and risk of breast cancer. JNCI, J. Natl. Cancer Inst. 1993, 85 (8), 648–652. 10.1093/jnci/85.8.648. 8468722

[ref45] Mekonen S.; Ibrahim M.; Astatkie H.; Abreha A. Exposure to organochlorine pesticides as a predictor to breast cancer: A case-control study among Ethiopian women. PLoS One 2021, 16 (9), e0257704 10.1371/journal.pone.0257704. 34555072 PMC8460037

[ref46] Garg P. K.; Chishi N.; Kumar R.; Latha T. K.; Rai S.; Banerjee B. D.; Gupta S. Organochlorine Pesticide Tissue Levels in Benign and Malignant Breast Disease: A Comparative Exploratory Study. J. Environ. Pathol. Toxicol. Oncol. 2021, 40 (1), 43–50. 10.1615/JEnvironPatholToxicolOncol.2020035783. 33639072

[ref47] Silva A. M. C.; Campos P. H. N.; Mattos I. E.; Hajat S.; Lacerda E. M.; Ferreira M. J. M. Environmental Exposure to Pesticides and Breast Cancer in a Region of Intensive Agribusiness Activity in Brazil: A Case-Control Study. Int. J. Environ. Res. Public Health 2019, 16 (20), 3951 10.3390/ijerph16203951. 31627286 PMC6843507

[ref48] Grau D.; Grau N.; Gascuel Q.; Paroissin C.; Stratonovitch C.; Lairon D.; Devault D. A.; Di Cristofaro J. Quantifiable urine glyphosate levels detected in 99% of the French population, with higher values in men, in younger people, and in farmers. Environ. Sci. Pollut. Res. 2022, 29 (22), 32882–32893. 10.1007/s11356-021-18110-0. PMC9072501 35018595

[ref49] Schoeters G.; Verheyen V. J.; Colles A.; Remy S.; Martin L. R.; Govarts E.; Nelen V.; Den Hond E.; De Decker A.; Franken C.; Loots I.; Coertjens D.; Morrens B.; Bastiaensen M.; Gys C.; Malarvannan G.; Covaci A.; Nawrot T.; De Henauw S.; Bellemans M.; Leermakers M.; Van Larebeke N.; Baeyens W.; Jacobs G.; Voorspoels S.; Nielsen F.; Bruckers L. Internal exposure of Flemish teenagers to environmental pollutants: Results of the Flemish Environment and Health Study 2016–2020 (FLEHS IV). Int. J. Hyg. Environ. Health 2022, 242, 113972 10.1016/j.ijheh.2022.113972. 35453051

[ref50] Makris K. C.; Efthymiou N.; Konstantinou C.; Anastasi E.; Schoeters G.; Kolossa-Gehring M.; Katsonouri A. Oxidative stress of glyphosate, AMPA and metabolites of pyrethroids and chlorpyrifos pesticides among primary school children in Cyprus. Environ. Res. 2022, 212, 113316 10.1016/j.envres.2022.113316. 35439459

[ref51] Cosemans C.; Van Larebeke N.; Janssen B. G.; Martens D. S.; Baeyens W.; Bruckers L.; Den Hond E.; Coertjens D.; Nelen V.; Schoeters G.; Hoppe H. W.; Wolfs E.; Smeets K.; Nawrot T. S.; Plusquin M. Glyphosate and AMPA exposure in relation to markers of biological aging in an adult population-based study. Int. J. Hyg. Environ. Health 2022, 240, 113895 10.1016/j.ijheh.2021.113895. 34883335

[ref52] Fook S. M.; Azevedo E. F.; Costa M. M.; Feitosa I. L.; Bragagnoli G.; Mariz S. R. Avaliação das intoxicações por domissanitários em uma cidade do Nordeste do Brasil [Poisoning with household cleaning products in a city in Northeast Brazil]. Cad. Saúde Pública 2013, 29 (5), 1041–1045. 10.1590/S0102-311X2013000500021. 23703009

[ref53] Thomas K. W.; Dosemeci M.; Hoppin J. A.; Sheldon L. S.; Croghan C. W.; Gordon S. M.; Jones M. L.; Reynolds S. J.; Raymer J. H.; Akland G. G.; Lynch C. F.; Knott C. E.; Sandler D. P.; Blair A. E.; Alavanja M. C. Urinary biomarker, dermal, and air measurement results for 2,4-D and chlorpyrifos farm applicators in the Agricultural Health Study. J. Exposure Sci. Environ. Epidemiol. 2010, 20 (2), 119–134. 10.1038/jes.2009.6. PMC3633453 19240759

[ref54] Eaton J. L.; Cathey A. L.; Fernandez J. A.; Watkins D. J.; Silver M. K.; Milne G. L.; Velez-Vega C.; Rosario Z.; Cordero J.; Alshawabkeh A.; Meeker J. D. The association between urinary glyphosate and aminomethyl phosphonic acid with biomarkers of oxidative stress among pregnant women in the PROTECT birth cohort study. Ecotoxicol. Environ. Saf. 2022, 233, 113300 10.1016/j.ecoenv.2022.113300. 35158254 PMC8920761

[ref55] Freisthler M. S.; Robbins C. R.; Benbrook C. M.; Young H. A.; Haas D. M.; Winchester P. D.; Perry M. J. Association between increasing agricultural use of 2,4-D and population biomarkers of exposure: findings from the National Health and Nutrition Examination Survey, 2001–2014. Environ. Health 2022, 21 (1), 23 10.1186/s12940-021-00815-x. 35139875 PMC8830015

[ref56] Franke A. A.; Li X.; Shvetsov Y. B.; Lai J. F. Pilot study on the urinary excretion of the glyphosate metabolite aminomethylphosphonic acid and breast cancer risk: The Multiethnic Cohort study. Environ. Pollut. 2021, 277, 116848 10.1016/j.envpol.2021.116848. 33714786 PMC8044054

[ref57] Duforestel M.; Nadaradjane A.; Bougras-Cartron G.; Briand J.; Olivier C.; Frenel J. S.; Vallette F. M.; Lelièvre S. A.; Cartron P. F. Glyphosate Primes Mammary Cells for Tumorigenesis by Reprogramming the Epigenome in a TET3-Dependent Manner. Front. Genet. 2019, 10, 885 10.3389/fgene.2019.00885. 31611907 PMC6777643

[ref58] Hokanson R.; Fudge R.; Chowdhary R.; Busbee D. Alteration of estrogen-regulated gene expression in human cells induced by the agricultural and horticultural herbicide glyphosate. Human Exp. Toxicol. 2007, 26 (9), 747–752. 10.1177/0960327107083453. 17984146

[ref59] Thongprakaisang S.; Thiantanawat A.; Rangkadilok N.; Suriyo T.; Satayavivad J. Glyphosate induces human breast cancer cells growth via estrogen receptors. Food Chem. Toxicol. 2013, 59, 129–136. 10.1016/j.fct.2013.05.057. 23756170

[ref60] Mills P. K.; Yang R. Breast cancer risk in Hispanic agricultural workers in California. Int. J. Occup. Environ. Health 2005, 11 (2), 123–131. 10.1179/oeh.2005.11.2.123. 15875887

[ref61] Mesnage R.; Brandsma I.; Moelijker N.; Zhang G.; Antoniou M. N. Genotoxicity evaluation of 2,4-D, dicamba and glyphosate alone or in combination with cell reporter assays for DNA damage, oxidative stress and unfolded protein response. Food Chem. Toxicol. 2021, 157, 112601 10.1016/j.fct.2021.112601. 34626751

[ref62] Harada Y.; Tanaka N.; Ichikawa M.; Kamijo Y.; Sugiyama E.; Gonzalez F. J.; Aoyama T. PPARα-dependent cholesterol/testosterone disruption in Leydig cells mediates 2,4-dichlorophenoxyacetic acid-induced testicular toxicity in mice. Arch. Toxicol. 2016, 90 (12), 3061–3071. 10.1007/s00204-016-1669-z. 26838045 PMC6334304

[ref63] Congur G. Monitoring of glyphosate-DNA interaction and synergistic genotoxic effect of glyphosate and 2,4-dichlorophenoxyacetic acid using an electrochemical biosensor. Environ. Pollut. 2021, 271, 116360 10.1016/j.envpol.2020.116360. 33412448

[ref64] Huang P.; Yang J.; Song Q. Atrazine affects phosphoprotein and protein expression in MCF-10A human breast epithelial cells. Int. J. Mol. Sci. 2014, 15 (10), 17806–17826. 10.3390/ijms151017806. 25275270 PMC4227191

[ref65] Lasserre J. P.; Fack F.; Revets D.; Planchon S.; Renaut J.; Hoffmann L.; Gutleb A. C.; Muller C. P.; Bohn T. Effects of the endocrine disruptors atrazine and PCB 153 on the protein expression of MCF-7 human cells. J. Proteome Res. 2009, 8 (12), 5485–5496. 10.1021/pr900480f. 19778091

[ref66] Lasserre J. P.; Fack F.; Serchi T.; Revets D.; Planchon S.; Renaut J.; Hoffmann L.; Gutleb A. C.; Muller C. P.; Bohn T. Atrazine and PCB 153 and their effects on the proteome of subcellular fractions of human MCF-7 cells. Biochim. Biophys. Acta, Proteins Proteomics 2012, 1824 (6), 833–841. 10.1016/j.bbapap.2012.03.014. 22516319

[ref67] Mcelroy J. A.; Gangnon R. E.; Newcomb P. A.; Kanarek M. S.; Anderson H. A.; Brook J. V.; Trentham-Dietz A.; Remington P. L. Risk of breast cancer for women living in rural areas from adult exposure to atrazine from well water in Wisconsin. J. Exposure Sci. Environ. Epidemiol. 2007, 17 (2), 207–214. 10.1038/sj.jes.7500511. 16823399

[ref68] Hopenhayn-Rich C.; Stump M. L.; Browning S. R. Regional assessment of atrazine exposure and incidence of breast and ovarian cancers in Kentucky. Arch. Environ. Contam. Toxicol. 2002, 42 (1), 127–136. 10.1007/s002440010300. 11706377

[ref69] Gammon D. W.; Aldous C. N.; Carr W. C. Jr; Sanborn J. R.; Pfeifer K. F. A risk assessment of atrazine use in California: human health and ecological aspects. Pest Manage. Sci. 2005, 61 (4), 331–355. 10.1002/ps.1000. 15655806

[ref70] Koual M.; Tomkiewicz C.; Cano-Sancho G.; Antignac J. P.; Bats A. S.; Coumoul X. Environmental chemicals, breast cancer progression and drug resistance. Environ. Health 2020, 19 (1), 117 10.1186/s12940-020-00670-2. 33203443 PMC7672852

[ref71] Chaffer C. L.; Weinberg R. A. A perspective on cancer cell metastasis. Science 2011, 331 (6024), 1559–1564. 10.1126/science.1203543. 21436443

[ref72] Chen J.; Liu J.; Wu S.; Liu W.; Xia Y.; Zhao J.; Yang Y.; Wang Y.; Peng Y.; Zhao S. Atrazine Promoted Epithelial Ovarian Cancer Cells Proliferation and Metastasis by Inducing Low Dose Reactive Oxygen Species (ROS). Iran J. Biotechnol. 2021, 19 (2), e2623 10.30498/IJB.2021.2623. 34435054 PMC8358173

[ref73] Peyre L.; Zucchini-Pascal N.; Rahmani R. Atrazine represses S100A4 gene expression and TPA-induced motility in HepG2 cells. Toxicol. In Vitro 2014, 28 (2), 156–163. 10.1016/j.tiv.2013.10.019. 24211529

[ref74] Diamantopoulou Z.; Castro-Giner F.; Schwab F. D.; Foerster C.; Saini M.; Budinjas S.; Strittmatter K.; Krol I.; Seifert B.; Heinzelmann-Schwarz V.; Kurzeder C.; Rochlitz C.; Vetter M.; Weber W. P.; Aceto N. The metastatic spread of breast cancer accelerates during sleep. Nature 2022, 607 (7917), 156–162. 10.1038/s41586-022-04875-y. 35732738

[ref75] Kongtip P.; Nankongnab N.; Kallayanatham N.; Pengpumkiat S.; Gore R.; Pundee R.; Konthonbut P.; Woskie S. R. Disruption of the Diurnal Cortisol Hormone Pattern by Pesticide Use in a Longitudinal Study of Farmers in Thailand. Ann. Work Exposures Health 2021, 65 (4), 406–417. 10.1093/annweh/wxaa124. PMC8628653 33604604

[ref76] da Silva J. C.; Scandolara T. B.; Kern R.; dos Santos Jaques H.; Malanowski J.; Alves F. M.; Rech D.; Silveira G. F.; Panis C. Occupational Exposure to Pesticides Affects Pivotal Immunologic Anti-Tumor Responses in Breast Cancer Women from the Intermediate Risk of Recurrence and Death. Cancers 2022, 14 (21), 5199 10.3390/cancers14215199. 36358618 PMC9655347

